# Romiplostim for the Emergency Management of Severe Immune Thrombocytopenia with Intracerebral Hemorrhage

**DOI:** 10.3389/fneur.2017.00737

**Published:** 2018-01-15

**Authors:** Romain Gellens, Sabrina Habchi, Sebastien Freppel, David Couret, Silvia Iacobelli

**Affiliations:** ^1^Neurocritical Care Unit, Centre Hospitalier Universitaire Sud Réunion, Saint-Pierre, France; ^2^Department of Neurosurgery, Centre Hospitalier Universitaire Sud Réunion, Saint-Pierre, France; ^3^INSERM, UMR 1188 Diabète athérothrombose Thérapies Réunion Océan Indien (DéTROI), plateforme CYROI, Sainte Clotilde, France; ^4^Centre d’Études Périnatales de l’Océan Indien (CEPOI) – EA 7388, Centre Hospitalier Universitaire Sud Réunion, Saint-Pierre, France; ^5^Pediatric and neonatal intensive care unit, Centre Hospitalier Universitaire Sud Réunion, Saint-Pierre, France

**Keywords:** immune thrombocytopenia, romiplostim, thrombopoietin receptor agonist, intracerebral hemorrhage, hemorrhagic stroke

## Abstract

Currently, we lack well-established guidelines for the emergency management of severe immune thrombocytopenia (ITP) with life-threatening bleeding. We now report the management of two patients with severe ITP, complicated by substantial cerebral hemorrhage, requiring urgent surgery due to refractory intracranial hypertension. To rapidly boost platelet counts (PCs), corticosteroids, intravenous immunoglobulin, and iterative platelet transfusions were given; all were ineffectual. Romiplostim, a thrombopoietin receptor agonist, was then administered as an “on demand therapy,” with the result that a rapid and sustained increase of PCs was achieved, thus allowing for postoperative hemostasis. Both patients recovered good neurological condition, suggesting the potential utility of romiplostim, in combined therapy, for the emergency management of severe ITP.

## Introduction

Intracerebral hemorrhage (ICH) is a rare (1.4% in adults, <1% in children) (1) but devastating complication of severe immune thrombocytopenia (ITP), with high mortality (up to 25%) and morbidity (25% of neurologic sequelae) (2). Currently, we lack well-established guidelines for the emergency management of severe ITP with ICH.

In 2009, an international working group (3) proposed a new definition of immune thrombocytopenic purpura, adjusted in 2011 by The American Society of Hematology (4). ITP, as newly defined, is an acquired disease characterized by a platelet count (PC) less than 100 × 10^9^/L mediated by immune system disruption. In primary ITP, thrombocytopenia is isolated and unrelated to any obvious etiology. Secondary ITP is considered to be the result of underlying disease (infection, autoimmunity, and neoplastic disorder), or drug exposure, and its evolution often correlates with management of the triggering factor. ITP can be divided into three stages: newly diagnosed (0–3 months), persistent (3–12 months duration), and chronic ITP (lasting >12 months). The degree of the severity of ITP should be assessed by bleeding symptoms rather than by PC. However, ITP-specific bleeding measurement tools are not yet validated by learned societies (1). In guidelines and consensus reports (3, 4), the term “severe ITP” is reserved for patients who have clinically relevant bleeding; that is to say a bleeding considered sufficient in magnitude either to mandate a treatment or which requires additional intervention, regardless of the PC.

In adults, treatment of ITP should only be considered if there is a high risk of bleeding, commonly defined by a PC < 30 × 10(9)/L or in the case of severe ITP (3–5). First-line treatment consists of corticosteroids and/or intravenous immunoglobulin (IVIg), effective in 70–90% of patients, with median times to response of 4–14 and 1–4 days, respectively (5). Patients not responding to the first-line treatment require second-line therapy including splenectomy or various immunosuppressive agents that are not always appropriate for critically ill. Therefore, “on demand therapy” designates any therapy used to temporarily increase the PC in cases of major bleeding or to safely perform invasive procedures (3, 5).

Immune thrombocytopenia pathogenesis is mediated by autoantibodies resulting in both accelerated circulating platelet destruction and impaired platelet production. The second mechanism has prompted the recent development of drugs such as thrombopoietin (TPO) growth factor, and, latterly, TPO-receptor agonists (6). Romiplostim is a second-generation TPO-R agonist whose efficacy and safety have been assessed in recent randomized trials, especially in chronic ITP (7, 8). The US Food and Drug Administration and the European Medicine Agency have approved romiplostim for adults with chronic ITP who are unresponsive to corticosteroids, IVIg, or splenectomy. The recent ITP management guidelines (4, 5) also recommend or suggest TPO-R agonists as a second-line therapy for adults at risk of bleeding regardless of their splenectomized status: romiplostim showing an 89% response rate in non-splenectomized patients (8). Neither guideline recommends the use of romiplostim in children because of their common, spontaneous, and rapid remission. However, several recent randomized trials in children support the safety and efficacy of romiplostim in chronic ITP, with an overall response rate comparable to adults (9).

Romiplostim was also proposed in the preoperative period of elective surgeries (10), and as “on demand therapy” for patients with severe mucocutaneous bleeding, in newly diagnosed or chronic ITP (11, 12). However, there are very few reports of its use in the setting of ICH. We now describe two cases (one adult and one child) illustrating the use of romiplostim as “on demand therapy” for hemorrhagic stroke causing intracranial hypertension, in the setting of primary or secondary ITP. We consider a PC > 100 × 10^9^/L as a complete response to treatment, which is also the target PC commonly accepted for ICH. We obtained written and informed consent from the participants to publish this report.

## Case 1

**Figure 1:**
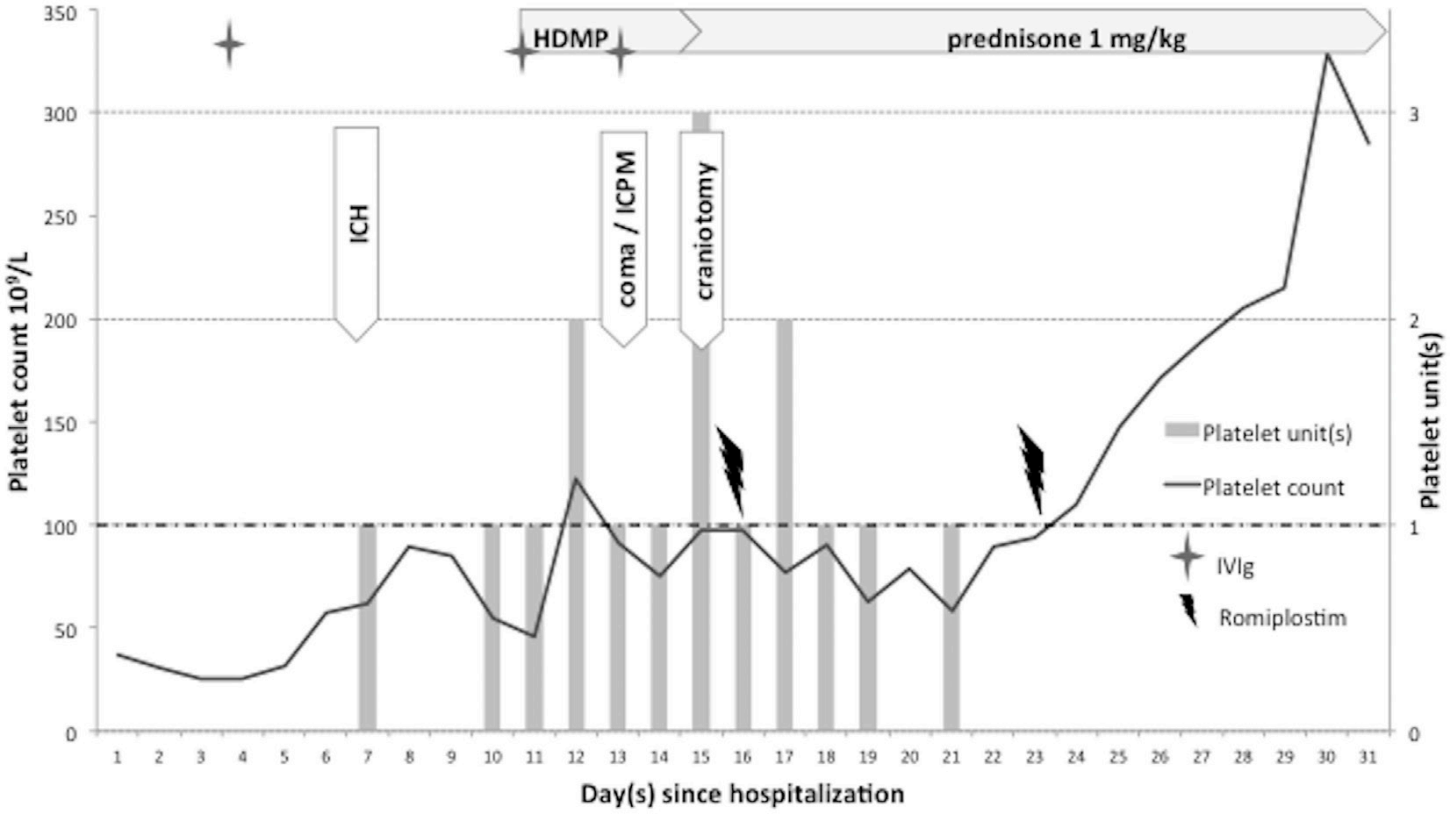
Patient 1. Platelet counts and platelet transfusions during intensive care hospitalization. Combined therapy including romiplostim as emergency management for severe immune thrombocytopenia complicated by intracranial hemorrhage (ICH). Intracranial pressure monitoring (ICPM); Romiplostim dose: 1 μg/kg subcutaneously; intravenous immunoglobulin (IVIg) dose: 1 g/kg; and high-dose methyl prednisolone (HDMP): 15 mg/kg/day.

**Figure 2:**
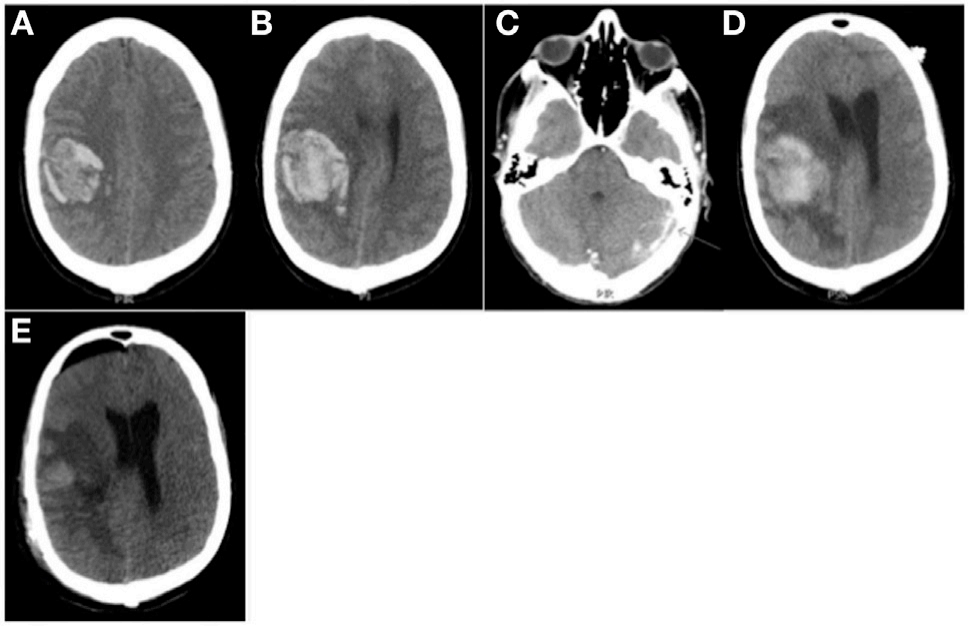
Patient 1. CT head **(A,B,D)** and CT venography **(C)** prior to surgery: **(A)** Day 6: large right frontal cerebral hematoma (40 mL), moderate mass effect with 4 mm of maximal brain midline shift. **(B)** Day 7: hematoma volume increase up to 45 mL. **(C)** Day 13: left lateral venous sinus thrombosis. **(D)** Day 14, just before surgery: increased mass effect (10 mm brain midline shift) with peripheral edema. CT head after surgery: **(E)** Day 16: decreased mass effect (6 mm brain midline shift).

A 29-year-old man, without a past medical history, was admitted in September 2015 at Réunion University Hospital for sepsis with eosinophilic pneumonia (Figures 1 and 2). His blood cell count revealed severe thrombocytopenia (31 × 10^9^/L) with hypereosinophilia (8 × 10^9^/L). Amoxicillin/clavulanic acid had been prescribed 1 week earlier for an inflammatory axillary tumefaction that occurred after shaving. The initial diagnostic workup revealed only pulmonary disorders: bronchoalveolar lavage showing marked eosinophilia (97%), alveolar hemorrhage, with quantitative culture of 10^4^ CFU/mL *Streptococcus oralis*. A bone marrow aspirate revealed rich marrow proliferation. Screens for autoimmune disease (including anti-phospholipids and ANCA vasculitis test) were negative; no allergies were reported. In the context of sepsis and a probable diagnosis of secondary ITP, the patient received a first course of IVIg, combined with antibiotics, and antiparasitics.

Six days after admission, the patient was transferred to the neurointensive care unit for both spontaneous intracerebral and abdominal hemorrhage. He presented with drowsiness, predominantly brachiofacial hemiplegia associated with lower back pain, and a PC of 60 × 10^9^/L. A CT scan showed a large hemorrhage in the right frontal lobe (40 mL) with mass effect (Figure 2A) and a large (6 cm) left adrenal gland hematoma. CT angiography revealed a spot sign within the brain hematoma without any suggestion of arteriovenous malformation or cerebral venous thrombosis. A 24-h follow-up CT scan revealed a slight expansion of the frontal hematoma (45 vs. 40 mL) (Figure 2B). Therefore, ITP therapy was intensified with iterative platelet transfusions to achieve a PC ≥ 100 × 10^9^/L.

At day 12, the patient was found to be in convulsive status epilepticus, and clinical seizure control was achieved with antiepileptic medication (benzodiazepine, then phenytoin). A sustainable decrease in Glasgow Coma Score to 8 required general anesthesia with IV midazolam and mechanical ventilation. An EEG revealed a non-convulsive status epilepticus controlled with propofol infusion. A brain CT venography showed a left lateral sinus thrombosis complicated by small temporal hemorrhage (Figure 2C). An intraparenchymal pressure monitor (Codman^®^ MicroSensor) was placed in the left frontal lobe; revealing an initial intracranial pressure (ICP) of 40 mmHg. ICP decreased only transiently despite deeper level of standard sedation (propofol, midazolam, and sufentanil), prevention of secondary systemic brain insults, pharmacological neuromuscular paralysis, and controlled mild hypothermia (35°C). Finally, a barbiturate coma was required to control intracranial hypertension but ICP increased again above 40 mmHg at day 14. A new CT scan showed no expansion of the right frontal hematoma but substantial worsening of edema with increased mass effect (Figure 2D). Because of refractory intracranial hypertension (ICHT), a mini-craniotomy was performed to evacuate the hematoma, although iterative platelet transfusions failed to secure the procedure and avoid perioperative bleeding. Thus, romiplostim was initiated, at the advice of the hematologist, with a first injection the day after surgery and a second course 1 week later. A sustainable complete response was achieved 8 days after first administering romiplostim. No further hemorrhage expansion or new bleeding into the craniotomy site was noted on follow-up brain imaging (Figure 2E). Note that immunosuppressive agents could not be used because of a concomitant septic shock related to a pyothorax.

Heparin could then be initiated for cerebral sinus thrombosis and further invasive procedures performed to complete the diagnostic workup (lung biopsy, transesophageal echocardiography, and cerebral angiogram), which remained non-contributory. Thereafter, the patient was discharged from hospital 2 months after being admitted with predominantly brachiofacial paresis.

## Case 2

**Figure 3:**
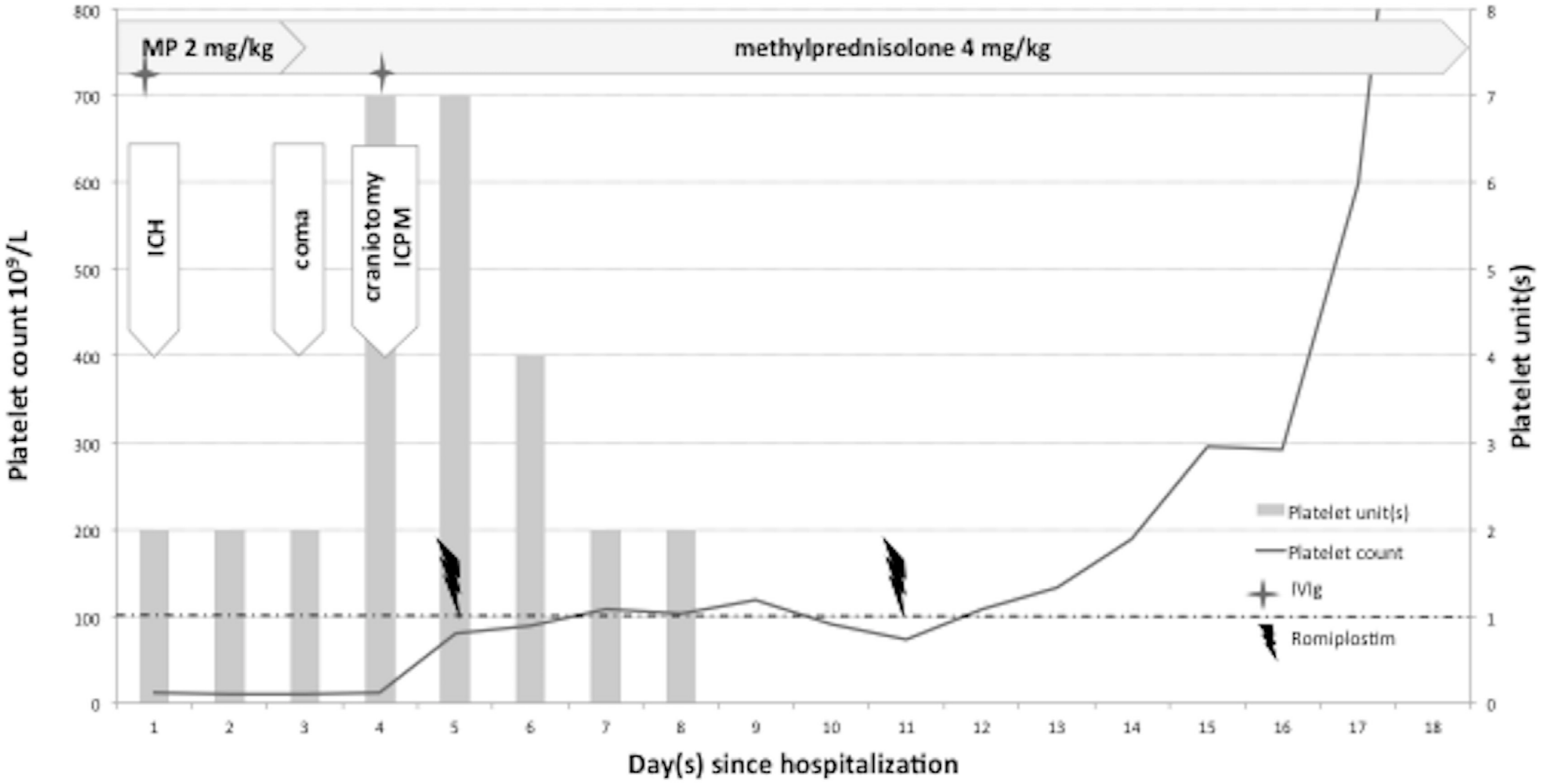
Patient 2. Platelet counts and platelet transfusions during intensive care hospitalization. Combined therapy including romiplostim as emergency management for severe ITP complicated by intracranial hemorrhage (ICH). ICPM = intracranial pressure monitoring (intraparenchymal probe). MP = methylprednisolone. Romiplostim dose: 10 μg/kg subcutaneously; intravenous immunoglobulin (IVIg) dose: 1 g/kg.

**Figure 4:**
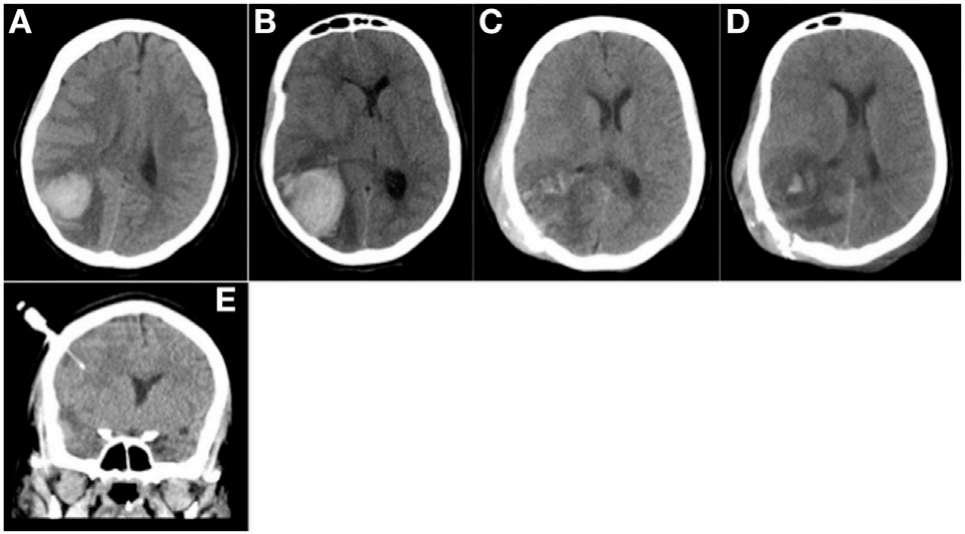
Patient 2. CT head **(A,B)** prior to surgery: **(A)** Day 1: 36 mL parieto-occipital hematoma with perilesional edema and slight mass effect. **(B)** Day 3: significant hematoma expansion (hematoma volume measured at 50 mL) and increased mass effect. CT head **(C–E)** after surgery: **(C)** Day 5; the day after surgery: hematoma evacuation; decreased mass effect; subcutaneous hematoma occurring during surgery. **(D)** Day 10: no rebleeding was noted. **(E)** Day 10: no hemorrhage was observed around the intraparenchymal probe (intracranial pressure monitoring).

A 14-year-old female teenager, with a previous medical history of chronic ITP (diagnosed in 2012), was admitted to Mayotte’s Hospital (French overseas department) in October 2015 for gingival bleeding, intense headaches, and drowsiness (Figures 3 and 4). A blood cell count revealed severe thrombocytopenia at 13 × 10^9^/L and hemoglobin of 80 g/L. A CT scan showed a 36 mL right parieto-occipital hematoma with significant perilesional edema and mass effect (Figure 4A). She received first-line therapy including corticosteroids, IVIg, and platelet transfusion. Subsequently, she had a generalized tonic–clonic seizure. On the third day, she was transferred to the Pediatric Intensive Care Unit of Saint Pierre (Reunion Island, France), which is equipped with a neurosurgical department.

After admission, the neurological status of the patient deteriorated, with coma and anisocoria related to cerebral hematoma growth (50 mL) (Figure 4B). A barbiturate-induced coma was required to control ICHT, and corticotherapy was intensified because of persistent severe thrombocytopenia. The day after, ICHT remained uncontrolled. Salvage surgery was performed (craniotomy with hematoma evacuation). Despite massive platelet transfusion in the perioperative period, the PC remained below 15 × 10^9^/L before the procedure, which was complicated by significant subcutaneous bleeding (Figure 4C). Consequently, romiplostim was initiated at the advice of the hematologist, with a first administration the day after surgery and a second course 1 week later. At this point, a PC of 81 × 10^9^/L was achieved, largely due to a massive platelet transfusion. A sustainable “complete response” was achieved only 6 days after romiplostim administration. We noted a PC peak of 1,554 × 10^9^/L 2 weeks after the second romiplostim injection, without clinical consequences. No further hemorrhage expansion or new bleeding into the craniotomy site was noted on follow-up brain imaging (Figure 4D). No hemorrhagic complication was observed related to the intraparenchymal ICP monitor (Figure 4E) placed at the end of the surgical procedure. ICP was initially measured at 27 mmHg and then decreased gradually by continuing sedation for a few more days.

Eighteen days after her hospitalization the patient was discharged from intensive care with left residual hemiparesis. She full recovered a few months later.

## Discussion

Hematoma volume and hematoma growth are key determinants of poor outcomes for ICH caused by hematologic disorders (13). Therefore, the aim of emergent management of ITP in the setting of ICH is to achieve a rapid and sufficient PC to stop bleeding, avoid hematoma growth, and permit operative intervention. Combination therapy (IVIg associated with corticosteroids and iterative platelet transfusions) appears to provide the most rapid rise in PC (4, 5) but may not be enough, especially in the setting of ICH where high PC level is required (80–100 × 10^9^/L), which leads to massive platelet transfusion. Critical illness (ICHT, sepsis) often limits the use of second-line ITP therapy. Our cases support the efficacy and safety of the emergency use of romiplostim to promptly restore a safe PC and secure the postoperative period in the setting of unresponsive and severe ITP with ICH. A sustainable complete PC response was obtained for both patients with a short course of romiplostim, and platelet transfusions were rapidly stopped. No significant side effects related to romiplostim (14) were reported despite many confounding factors due to the critical illness: the thrombosis in case 1 pre-existed romiplostim administration, with no rebound of deteriorating thrombocytopenia following romiplostim cessation.

The shorter time to response noted in our experience using romiplostim to achieve a PC > 100 × 10^9^/L compared to that reported in the Contis study (11) (~7 days vs. ~2 weeks) may be explained by our early initiation of romiplostim treatment after corticosteroids and IVIg (<7 days vs. several weeks). Given the need to reduce the time to response of ITP treatment to prevent cerebral hematoma growth, romiplostim could be associated with the combined first-line therapy for ITP patients with ICH and as much as possible before surgical procedure.

In the setting of combined ITP therapy, our experience suggests that timely neurosurgical intervention appears to be an acceptable risk as previously reported (15). Note that none of our patients experienced hemorrhagic complications after intracranial pressure monitor insertion (intraparenchymal probes were placed after platelet transfusion).

The doses used in our cases are those usually recommended in the literature but correspond to extreme values (range from 1 to 10 µg/kg weekly) (6–9). Indeed, the usual recommended starting dose is 3–6 μg/kg/weekly. The intensity of the PC response appears to be related to a “dose effect” of romiplostim as previously reported (6).

Significant sparing of platelet transfusions contributes to the cost-effectiveness of romiplostim (16). Indeed, to achieve a PC of 100 × 10^9^/L, our patients received 16 and 28 platelet concentrates, the majority before romiplostim administration. In fact, previous work suggests that romiplostim leads to bleeding cessation before the PC increases. This could be related to an activation of pre-existing platelets and a restoration of immune tolerance to platelet antigens (14).

In conclusion, our cases support the potential utility of a combined therapy including romiplostim, for the emergent management of ICH in the setting of primary or secondary ITP in adults and pediatric patients.

## Ethics Statement

Case report: patient consents are in attached files.

## Author Contributions

RG: conception and design, acquisition of data, drafting the article, reviewed submitted version of manuscript, approved the final version of the manuscript on behalf of all authors, administrative/technical/material support, and study supervision. SH: conception and design, drafting the article, critically revising the article, reviewed submitted version of manuscript, and study supervision. SF: critically revising the article and reviewed submitted version of manuscript. DC: conception and design, critically revising the article, reviewed submitted version of manuscript, approved the final version of the manuscript, and study supervision. SI: conception and design, acquisition of data, critically revising the article, reviewed submitted version of manuscript, approved the final version of the manuscript, administrative/technical/material support and study supervision.

## Conflict of Interest Statement

The authors declare that the research was conducted in the absence of any commercial or financial relationships that could be construed as a potential conflict of interest.
